# A Systematic Review of Arsenic Exposure and Its Social and Mental Health Effects with Special Reference to Bangladesh

**DOI:** 10.3390/ijerph6051609

**Published:** 2009-05-08

**Authors:** Johanna Brinkel, Mobarak H. Khan, Alexander Kraemer

**Affiliations:** Department of Public Health Medicine, University of Bielefeld, Bielefeld, Germany; E-Mails: johanna_katharina.brinkel@uni-bielefeld.de (J.B.); alexander.kraemer@uni-bielefeld.de (A.K.)

**Keywords:** Arsenic, mental health, social problems, Bangladesh

## Abstract

Underground water in many regions of the world is contaminated with high concentrations of arsenic and the resulting toxicity has created a major environmental and public health problem in the affected regions. Chronic arsenic exposure can cause many diseases, including various physical and psychological harms. Although the physical problems caused by arsenic toxicity are well reported in literature, unfortunately the consequences of arsenic exposure on mental health are not adequately studied. Therefore we conducted a review of the available literature focusing on the social consequences and detrimental effects of arsenic toxicity on mental health. Chronic arsenic exposures have serious implications for its victims (i.e. arsenicosis patients) and their families including social instability, social discrimination, refusal of victims by community and families, and marriage-related problems. Some studies conducted in arsenic affected areas revealed that arsenic exposures are associated with various neurologic problems. Chronic arsenic exposure can lead to mental retardation and developmental disabilities such as physical, cognitive, psychological, sensory and speech impairments. As health is defined by the World Health Organization as “a state of complete physical, mental and social wellbeing”, the social dimensions have a large impact on individual’s mental health. Furthermore studies in China und Bangladesh have shown that mental health problems (e.g. depression) are more common among the people affected by arsenic contamination. Our study indicates various neurological, mental and social consequences among arsenic affected victims. Further studies are recommended in arsenic-affected areas to understand the underlying mechanisms of poor mental health caused by arsenic exposure.

## 1. Introduction

Arsenic (As) is one of the oldest poisons known to men and its applications throughout history are wide and varied [1]. Because arsenic in the bedrock is easily dissolved into surrounding water, inorganic arsenic is frequently present at elevated concentrations in groundwater [2,3]. The catastrophe of arsenic toxicity, caused by arsenic contaminated water, has already been reported in many countries of the world, namely in Bangladesh, India, Nepal, Cambodia, Myanmar, Taiwan, Mongolia, Vietnam, China, Afghanistan, Pakistan, Argentina, Mexico, Chile and the United States [4–8]. Based on the above-mentioned information, the Asian region is much more affected by arsenic, as compared to other regions of the world. Unfortunately, as a single country Bangladesh has experienced the largest arsenic catastrophe in human history [5]. According to the recommendations of the World Health Organization, the maximum permissible level of arsenic in drinking water is 0.05 mg/L in Bangladesh, although this limit is much lower (≤0.01 mg/L) for many other countries [9,10].

Bangladesh has an abundance of surface and underground water because it is mostly a flat deltaic land formed by the mechanical action of the great Himalayan rivers - the Ganges, Brahmaputra and Meghna [11]. Unfortunately in Bangladesh these sources are highly contaminated and pose a great threat to the nation, particularly from the public health point of view. The surface water is contaminated by many pollutants (e.g. originated from industrial activities, household activities, human waste, and poor environmental and garbage management) including micro-organisms related to water-borne diseases. To reduce the burden of water-borne diseases in Bangladesh, the majority of people drink the pathogen free underground water. For instance, about 97% of the rural population drinks underground water through millions of hand-pump tubewells. Unfortunately the underground water of Bangladesh is now widely contaminated by arsenic (>0.05 mg/L). For instance, a higher concentration of arsenic was identified in 61 out of 64 districts and about 29% of the total tubewells was arsenic contaminated [12]. Based on estimation, about 35 million and 57 million inhabitants in Bangladesh are at risk of drinking arsenic contaminated water exceeding 0.05 mg/L and 0.01 mg/L, respectively [4].

Drinking arsenic-contaminated water for a long time is toxic and affects all organs and systems of the body [10,13]. Furthermore sufferers of arsenicosis develop cardiovascular, hepatic, renal, gastrointestinal, neurological and reproductive problems and malignancies [12]. The most common manifestations among arsenicosis patients are skin lesions [5]. Besides these problems, arsenicosis diseases may cause psychological harms and affect mental health [14]. However, the effects of arsenic toxicity on mental health and associated social consequences have not been well reported and hence more scientific attention is needed. The primary objective of this review is to report the effects of chronic arsenic exposure on mental health and associated social consequences of arsenic victims. To our knowledge, this is the first article to address such a topic in Bangladesh.

## 2. Methods

We conducted this systematic review using electronic databases to report on arsenic-related mental health and social consequences. To find relevant studies we used various databases, including *PubMed, PsycINFO* and *the Cochrane Library.* We also accessed to numerous library catalogues, subject-specific databases and international catalogues, including the databases *Arts and Humanities Citation Index (A&HCI)*, article database *Jade*, *Science Citation Index Expanded (SCI-E)* and *Social Sciences Citation Index (SSCI)* and various journals available at the University of Bielefeld Library. The database of the National Institute of Mental Health (NIMH) and the section of the Occupational and Safety Hazard Administration (OSHA) website (part of the U.S. Department of Labour, Washington) were also used. Additionally we contacted the NIMH, which is an expert institution in the field of mental health, to get relevant studies/information which meets the inclusion criteria of the review.

Relevant studies have been searched by using the following key words: *arsenic* as a single word or arsenic* or *arsenicosis* and *“mental health*”, or depress*, or “mental defect*”, or *“well-being*”, or psychosis*, or social*, social conclusion*, social hazard*, social influnce*, brain* function*, neurotoxicologi* effect*.

We included all types of relevant studies like journal articles, reports and book chapters into our review, because of limited information regarding our topic of interest. Moreover, our research question could be answered by any type of study. Initially we read all the abstracts to judge the suitability of the studies and then all the suitable references were added to the list of articles. Next we retrieved and saved the full texts of the selected references. We also collected some studies by interlibrary lending. Later we again assessed the contents of the literatures and rated them according to mental health and social risk and social hazard items. A total of 66 articles were assessed, of which 44 articles were finally used in this review (please see the graph below). We excluded those articles which were not written in English and not context-specific.

## 3. Results and Discussion

This disease creates enormous social problems like social hazards, poverty, social instability, superstition, ostracism, and marriage related problems. It is also associated with poor mental health and developmental disabilities. These issues based on various studies are discussed below.

### 3.1. Social Hazards and Poverty

Arsenic is not only a physical but also a social phenomenon [9]. Besides arsenic toxicity and arsenicosis diseases, arsenic poisoning creates extensive social implications for its victims and their families in affected areas. A number of socio-economic problems like social uncertainty, social injustice, social isolation and problematic family issues are reported due to arsenicosis [15,16]. Arsenicosis is found to be more prevalent among the poor [12,17–19] who suffer from dietary deficiency, who have no alternative sources of safe drinking water and who are unable to get proper care and treatment because of financial constraints. The effects of long-term arsenicosis are also severe in the poor and could cause social problems, interrupt the societal ties [18] and trigger the social problems. Unfortunately, these social problems in affected areas are still not fully recognized and understood [18].

There is a strong link between poverty and arsenicosis diseases. Arsenicosis enhances the economic burden of the poor. The majority of victims are considered as a burden to their family and society. Most of the poor arsenicosis patients remain untreated due to financial restraints. For example, 20–70% of the patients did not receive any treatment in Bangladesh due to financial problems [18]. This lack of treatment further deteriorates the overall health and economic conditions of arsenicosis victims. Because poverty rises as the untreated poor victims are incapable of doing hard work and gradually lose strength to move. This disease is associated with social discriminations such as losing jobs, barriers to access new jobs and social rejections. Again, if the poor arsenicosis patients go for treatment, they need to spend a big proportion of their money on this, which finally diminishes the household income and increases the economic burden on the poor victims and their families [12]. Moreover, the cost of obtaning arsenic free water also diminishes household income [18].

### 3.2. Social Instability

Chowdhury *et al.* [18] and Nasreen [9] have described the extreme instability of social life in Bangladesh due to arsenicosis. Arsenic is producing social stigmatization and discrimination [18]. Unaffected people are generally scared of arsenicosis, therefore they tend to avoid and isolate arsenic victims [9,18,20,21]. Social conflicts over contaminated water destroy the social harmony and network relationships [9,18]. Arsenic victims are often wrongly identified as leprosy patients and isolated from their close relations [22]. In many cases the victims attribute their diseases to their fate [23]. Arsenicosis disease hampers socialization by social stigmatization and discrimination [9]. For instance, arsenic patients often remain ostracized in all age-groups and barred from social activities. Children of arsenicosis patients are not allowed to attend social and religious functions as well as denied to take water from a neighbour’s tube well and students debarred from school [9,24]. Affected families are also not allowed to take baths in any of the village ponds [9]. Some unaffected people behave in a hostile manner and think that patients should either stay in their homes or leave the village [15].

### 3.3. Ostracism and Marriage Related Problems

Arsenic victims are abandoned, not only by society but also by their family members. There are some instances that arsenicosis leads to a break-down of the marital relationships. For instance, wives were divorced or separated or sent back to their parents’ house because of the arsenicosis disease [18,24]. There are also some evidences that wives left arsenic affected husbands because they were afraid of arsenicosis [9]. Problems before marriage are also notable. For example, it is difficult to find a spouse for an arsenic victim. Generally people are reluctant to establish marital relationships with those families suffering from arsenicosis. Young women and men in the affected families are advised to remain unmarried [18]. Such incidents cause unlimited anxiety for both patients and parents of arsenic-affected adult children [9,18]. In Bangladesh, arsenicosis women are the worst victims of ostracism than arsenicosis men, because they are vulnerable by two ways: firstly by the disease itself and secondly by becoming outcast. Affected women also experience socially undesirable events like dowry, physical torture, and polygamy [18]. Due to the patriarchal system and lower socio-cultural position of women in the society, unmarried women and women abandoned by husband and families live inhumanly [9,18].

### 3.4. Superstition

Public awareness is necessary to fight against arsenicosis [18]. Unfortunately poor people living in rural areas of Bangladesh are not adequately informed about arsenic contamination and arsenicosis [9,12,15,25,26]. As a result of ignorance, mainly among the illiterate people in the remote villages, some people believe in superstitions, prejudices and fairy tales [9,18]. For instance, some people think that the disease is ‘an act of the devil/impure air’ or ‘a curse of God’ or ‘the work of evil spirits’ [9,18]. Due to such superstitions and prejudices, 30 to 80% of the patients in an arsenic affected village in Bangladesh did not receive any treatment [18].

### 3.5. Neurotoxicological Effects

Areas of neurotoxicological impairments include poor cognitive performance and disturbances in visual perception, psychomotor speed, attention, speech and memory [27]. Various studies have reported the neurologic sequelae of acute and chronic arsenic exposure in adults and children [28–33]. Various studies [34–37] also confirmed that some chemical toxicants have the potential to modify brain physiology and can lead to mental retardation and developmental disabilities such as physical, cognitive, psychological, sensory and speech impairments including socially undesirable outcomes (also see Table 1). Two studies conducted in an arsenic affected area of Bangladesh showed that exposure to arsenic from drinking water was associated with reduced intellectual function (such as performance and processing speed) in a dose-response manner even after adjusting the impact of important sociodemographic covariates [31,33]. The study of Calderon *et al.* [29] in Mexico (n=80) found negative relationships between children’s urinary arsenic and verbal intelligence after controlling for a small set of demographic factors. Another recent study based on Mexican schoolchildren reported significantly inverse associations between urinary arsenic and visual-spatial abilities (cognitive performance) such as the Peabody picture vocabulary test, visual search, and letter sequencing tests by a covariate-adjusted model [27]. Comparing high school students from an arsenic-affected area with a non-affected area in Taiwan (n=109), poorer scores of neurobehavioral performance were found in 3 of 4 tests in adolescents from the arsenic affected area [30]. There are some other studies which also confirmed the detrimental effects of arsenic on children intellectual functions [32,38,39]. The underlying mechanism is not yet clear but the ATSDR [36] stated that perhaps chronic long-term exposure to arsenic may intefere with neurotransmitters associated with depressions. Arsenic crosses the blood-brain barrier and has a wide range of effects on the white matter in the brain. Arsenic inhibits the synthesis and liberation of acetylcholine in brain slices and increases the monoamine activity in nervous system [27]. Decreased locomotors activity and oxidative stress reactions due to arsenic toxicity may also affect central nervous system [32]. Cognitive impairment was reported in two workers in the USA and mental functions returned to normal after withdrawal from arsenic [28].

### 3.6. Mental Health

As health is defined by the World Health Organization as “a state of complete physical, mental and social wellbeing” [2], therefore the mentioned psychical and social dimensions have a large impact on individual’s mental health. Chronic illness affects all kind of activities, including the activities of daily living, which deteriorates the quality of life of the victims, as well as subjective well-being and mental health [24]. Therefore persons with chronic illness are often in a state of crisis marked by physical, social and mental adverse effects. Taylor and Aspinwall [40] have described some common feelings like feelings of fear, anxiety and disorganization due to chronic diseases.

According to the study of Havenaar and van den Brink [14], exposure to toxic substances is not only causing physical harms but also psychological harms Although there are few studies available regarding arsenic exposure and mental health in literature, the mental health burden caused by arsenic poisoning seems to be remarkable. For instance, 36% of the arsenic victims (n=63) were suffering from a full or partial Post-traumatic Stress Disorder (PTSD) after the arsenic poisoning in 1998 in an arsenic affected community of West Japan [41]. Although Bangladesh is the most arsenic-affected country in the world, only one study has focused on mental health among arsenic victims (n=147) living in Rajarampur area of Chapainawabganj district (one of the arsenic hotspots) in Bangladesh [24]. According to this study, mental health scores differed significantly between arsenic-affected and healthy people, with poorer mental health (based on General Health Questionnaire 12) among arsenic-affected people [24]. Employing a case-control study in an arsenic-affected rural area in Bangladesh, Khan *et al.* [12] also found significantly higher level of depression, weakness, restlessness, insufficient sleep, drowsiness and loss of appetite among the arsenicosis cases as compared to controls. Around 61% of the cases and only around 44% percent of the controls reported depressive feelings with p-value= 0.011. In the same study, the authors also found significant associations between chronic arsenic exposure and subjective symptoms, such as fatigue and gastrointestinal symptoms [12] which might reflect mental health problems.

Besides the above-mentioned studies in Bangladesh, a study in the United States reported that people with higher arsenic contamination suffered more from depression [42]. The study investigated arsenic exposure and nine self-reported chronic diseases, including depression from a total of 1185 people. Individuals with wells in the mid-strata of arsenic concentrations (between 2 ug/L and 10 ug/L) were significantly more likely to report depression than individuals in the lowest strata (arsenic concentration <2 ug/L) in the United States (adjusted OR=2.74; 95% CI=1.14–6.63) [42]. Furthermore, a cross-sectional study in two villages in Inner Mongolia, China found that the mental health of the subjects in the arsenic-affected village was worse than in those in the arsenic-free village (OR= 2.5, 95% CI=1.1.–6.0) [43]. In addition, experiences with animals have pointed out that perinatal arsenic exposure was associated with depressive-like behaviors in the affected mouse offspring [44].

## 4. Conclusions

This review provides an overview of the mental health problem and social consequences among the arsenic-affected victims. The huge effects of arsenic on physical and mental health make the arsenic issue a most important public health issue particularly in Bangladesh. According to the World Health Organization, health is not only the absence of disease but the state of complete physical, mental and social well-being. Therefore, both physical and social conditions of the arsenic victims can affect their mental health. Although many studies have already been done regarding physical health outcomes, studies on the association between arsenic and mental health are lacking. The effects of arsenic on mental health deserve further investigation to protect the victims’ mental health and to enhance their psychological well being. This is important because arsenic victims are living with social uncertainty, social injustice, social isolation and problematic family issues. Arsenic can also cause neurotoxicological problems, which often leads to changes in behaviour.

Some of the limitations of this study were as follows: we included only those studies (articles, book chapters and reports) which were written in English. Some of the studies were based on small sample sizes [e.g. 24,28–30,41]. We did not put any restriction on study design while selecting our studies. As a result, we used studies like ecological [e.g. 42], case-control [e.g. 12,19,38,43,44], cohort [e.g. 19,38], and cross-sectional [e.g. 27,33,39] and case-study [e.g. 28] in our review. Studies [12,42] based on self-reported illness may also cause some potential bias.

Sensitization of community members and law enforcement authority to prevent separation and ostracism may be helpful to improve mental health of the arsenic victims. Rehabilitation programs for arsenicosis patients especially for women are needed. To overcome the socio-economic crisis, psychosocial support and employment opportunities should be provided to the patients. Victims need accurate health information as well as supportive counseling to improve their stress situation. Finally, the mental health burden in arsenic-affected areas should be estimated by larger studies and considered in the wider context of public and community health to understand the underlying mechanism of poor mental health due to arsenic and arsenicosis.

## Figures and Tables

**Figure 1.**
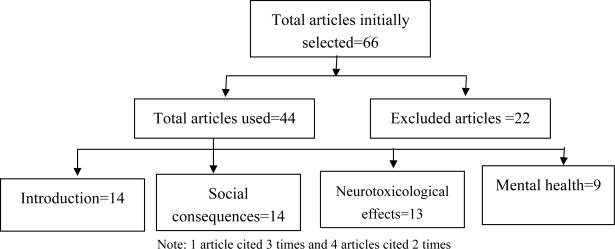
Literature-mapping for the review. Note: 1 article cited 3 times and 4 articles cited 2 times

**Table 1.** Neurotoxicological effects due to arsenic exposure among children in arsenic affected countries.

Authors and years of publication	Sample size (target population) and study design	Main results
Rosado *et al.* [27].	n=602 (6–8 years old school children in Mexico); Cross-sectional	Arsenic affected children’s cognitive development after adjusting for age, sex, mother’s education and hemoglobin concentration and so on.
Wasserman *et al.* [33].	n=301 (6 years old children in Bangladesh); Cross-sectional	Arsenic exposure was negatively associated with children intellectual level after adjusting for many potential variables.
Wasserman *et al.* [31].	n=201 (10 years old children in Bangladesh); Cross-sectional	Arsenic exposure was negatively associated with children intellectual level after adjusting for many potential variables.
Tsai *et al.* [30].	n=109 (School adolescents with an average age of 14 years in Taiwan); Case-control	Neurobehavioral development like pattern memory and switching attention were significantly affected by long-term exposure to arsenic after adjusting for education and sex.
von Ehrenstein *et al. [32].*	n=351 (5 to 15 years old children from the source population in West Bengal, India); Cross-sectional	Current arsenic concentrations in urine were associated with small decrements in intellectual testing in school-aged children.
Calderon *et al.* [29].	n=80 (6 to 9 years School children in Mexico); Cross-sectional	Higher level of urinary arsenic had negative influences on CNS function like verbal comprehension, long-term memory and attention.
Asadullah and Chaudhury [39].	n=7,710 (Secondary school children (enrolled in grade 8) in Bangladesh); Cross-sectional	Cognitive development of children is significantly negatively affected by arsenic.
